# Contribution of Impaired Insulin Signaling to the Pathogenesis of Diabetic Cardiomyopathy

**DOI:** 10.3390/ijms20112833

**Published:** 2019-06-11

**Authors:** Mònica Zamora, Josep A. Villena

**Affiliations:** 1 BCNatal, Fetal Medicine Research Center, Hospital Clínic and Hospital Sant Joan de Déu, University of Barcelona, 08950 Barcelona, Spain; MZAMORAV@hospitalclinic.org; 2 Institut d’Investigacions Biomèdiques August Pi i Sunyer (IDIBAPS), 08036 Barcelona, Spain; 3 Laboratory of Metabolism and Obesity, Vall d’Hebron—Institut de Recerca, Universitat Autònoma de Barcelona, 08035 Barcelona, Spain; 4 CIBERDEM, CIBER on Diabetes and Associated Metabolic Diseases, Instituto de Salud Carlos III, 28029 Madrid, Spain

**Keywords:** insulin resistance, diabetes, heart, diabetic cardiomyopathy, metabolic flexibility

## Abstract

Diabetic cardiomyopathy (DCM) has emerged as a relevant cause of heart failure among the diabetic population. Defined as a cardiac dysfunction that develops in diabetic patients independently of other major cardiovascular risks factors, such as high blood pressure and coronary artery disease, the underlying cause of DCMremains to be unveiled. Several pathogenic factors, including glucose and lipid toxicity, mitochondrial dysfunction, increased oxidative stress, sustained activation of the renin-angiotensin system (RAS) or altered calcium homeostasis, have been shown to contribute to the structural and functional alterations that characterize diabetic hearts. However, all these pathogenic mechanisms appear to stem from the metabolic inflexibility imposed by insulin resistance or lack of insulin signaling. This results in absolute reliance on fatty acids for the synthesis of ATP and impairment of glucose oxidation. Glucose is then rerouted to other metabolic pathways, with harmful effects on cardiomyocyte function. Here, we discuss the role that impaired cardiac insulin signaling in diabetic or insulin-resistant individuals plays in the onset and progression of DCM.

## 1. Introduction

Diabetes has become the most common metabolic disorder in the world and is nowadays recognized by the World Health Organization as one of the most deadly non-communicable diseases worldwide [1]. Cardiovascular diseases, including heart failure, ischemic heart, and stroke, lead the ranking of causes of death among the diabetic population. Decades ago, the Framingham study already showed that diabetes increases the risk of heart failure by two- and five-fold in men and women, respectively [2]. The elevated rates of heart failure in diabetic patients could be attributed, in part, to hypertension and atherosclerosis, two conditions that appear in diabetic patients with higher frequency than in the non-diabetic population. In support of a causal relationship between diabetes and cardiovascular diseases, it has been reported that an increase by 1% in the circulating levels of HbA1c is associated with 8% increase in the risk of cardiovascular disease in type 2 diabetic patients, even after adjusting for age, sex, obesity, alcohol, and tobacco consumption, hypertension or type and duration of diabetes [3]. The risk increases by up to 30% among the type 1 diabetic population [4]. Conversely, 1% reduction in the HbA1c is associated with a 16% lower risk of developing chronic heart failure [5]. These data support the notion that diabetes and the pathophysiologic alterations associated with this endocrine disease represent a unique and independent risk factor for the development of cardiac dysfunction. In fact, it is now recognized that heart failure in diabetic individuals can also be the consequence of diabetic cardiomyopathy (DCM), a pathological condition originally described by Rubler and collaborators in 1972 that is defined as a heart failure syndrome appearing in diabetic patients in the absence of coronary artery disease and high blood pressure [6]. However, the mechanistic basis of the development of DCM is still poorly understood.

Loss or attenuation of insulin signaling is a distinctive trait of diabetes. Whereas lack of insulin synthesis and/or secretion defines type 1 diabetes, tissue resistance to the action of insulin constitutes the hallmark of type 2 diabetes, which represents more than 90% of the cases of diabetes. Insulin resistance is considered the primary cause of type 2 diabetes. Although the metabolic disturbances and cellular alterations that insulin resistance causes in major insulin target tissues, such as muscle, liver or adipose tissue are well defined, less is known about the effects that insulin resistance has on cardiac function. In this review, we summarize the current knowledge about the role that impaired insulin signaling in heart plays in the pathogenesis of DCM.

## 2. Epidemiology and Pathophysiology of DCM

Even though increasingly recognized as a distinct pathological entity, only a few studies have been conducted to estimate the prevalence of DCM within the diabetic population. Due to the small number of participants and the disparity of the non-invasive imaging techniques used for the diagnosis of DCM, such studies have yielded very variable prevalence rates of DCM, ranging from 30% to 60%, depending on the study [7,8,9].

DCM is characterized by functional and structural changes in the heart of diabetic patients. At the functional level, patients with DCM exhibit early development of left ventricular diastolic dysfunction, featured by a reduction in the left ventricle filling velocity and delayed relaxation pattern. Left ventricle systolic dysfunction, being recognized by diminished ejection fraction, develops later as the disease progresses. Patients with DCM also show left ventricular hypertrophy, often accompanied by fibrosis. Interstitial fibrosis increases in parallel to the progression of the disease as a result of the replacement with the connective tissue of cardiomyocytes that have died from apoptosis and/or necrosis [10,11]. The developed fibrosis contributes to cardiac stiffness and impaired contractility of the heart. Another common feature of the heart of patients with DCM is cardiac steatosis [11], an abnormal accumulation of triglycerides in the myocardium that reflects the metabolic derangements occurring in cardiomyocytes of DCM patients (discussed in Section 3.1.3).

## 3. Insulin Resistance and the Pathogenesis of DCM

Established DCM is characterized by the clinical symptomatology described in the previous paragraph. However, DCM has a long subclinical course before the appearance of the first overt cardiac contractile and structural alterations, precluding an early detection and treatment of the disease. A fundamental question is how diabetes alters proper cardiomyocyte function and leads to the cardiac alterations that define DCM?

**Figure 1:**
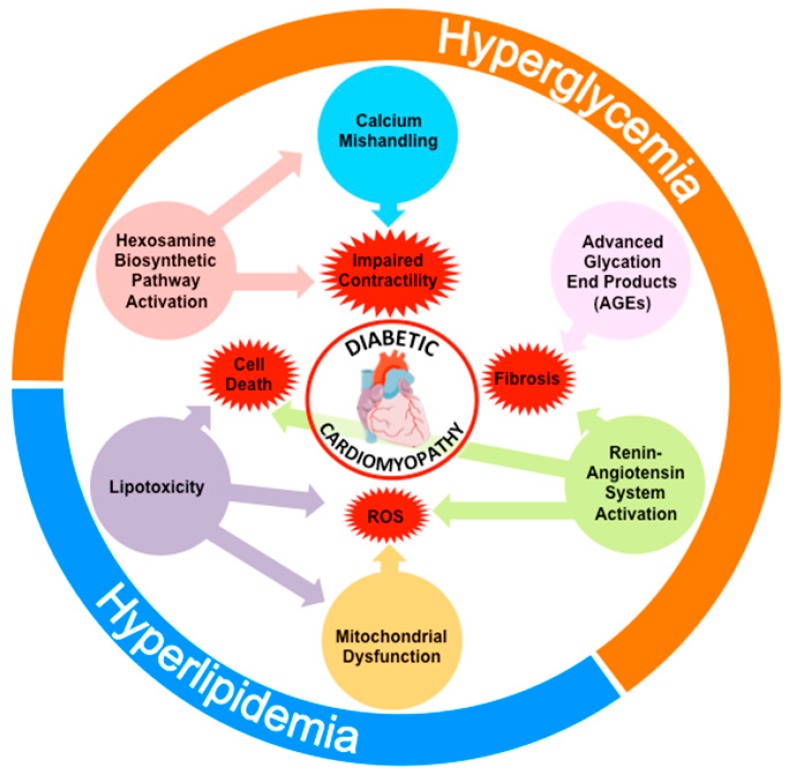
Overview of the molecular derangements linked to the pathogenesis of DCM. As a result of the metabolic inflexibility imposed by impaired insulin signaling, glucose is not oxidized by cardiomyocytes but instead is diverted into other metabolic pathways that lead to the synthesis of AGEsor to the *O*-GlyNacylation of proteins. The increased intracellular accumulation of glucose also activates the RAS pathway and alters calcium homeostasis. In addition, hyperlipidemia facilitates fatty acid uptake and leads to lipotoxicity and cardiac steatosis. Furthermore, enhanced but inefficient oxidation of fatty acids leads to the production of reactive oxygen species (ROS) and further promotes mitochondrial dysfunction. All these factors have been shown to contribute to some extent to the functional alterations that characterize DCM.

Numerous cellular and molecular factors have been proposed to play a causative role in the cardiac functional defects found in patients with DCM. This includes excessive production of advanced glycation end-products (AGEs), activation of the hexosamine biosynthetic pathway, lipotoxicity, mitochondrial dysfunction, increased oxidative stress, activation of the RAS or impaired calcium homeostasis (Figure 1). All these processes often concur in the same diabetic individual and interact among each other to exacerbate the structural and functional alterations of the diabetic heart. Despite their different nature and mechanisms of action, most of these pathogenic factors appear to stem from the same fundamental process commonly altered in diabetic patients: Impaired insulin signaling. Consequently, the reduction in the cardiac action of insulin could be considered as the primary cause of DCM.

Although its role may seem obvious, the precise contribution of altered insulin signaling to the cardiac alterations in humans has not been comprehensively studied yet, and it remains controversial. In fact, some studies have reported a failure to find insulin resistance in hearts of the same type 2 diabetic patients in which other insulin target tissues, such as skeletal muscle or adipose tissue, exhibit a clear resistance to the action of the hormone [12,13]. In such studies, it has been speculated that, in the absence of cardiac insulin resistance, the hyperinsulinemia that accompanies type 2 diabetes at some point along the progression of the disease could contribute to cardiomyocyte hypertrophy, given the known action of insulin as an anabolic hormone involved in cell growth. Nevertheless, numerous studies in animal models clearly support the notion that cardiac insulin resistance does exist in the context of type 2 diabetes or obesity with whole-body insulin resistance [14,15,16].

### 3.1. Molecular and Cellular Mechanisms of DCM

#### 3.1.1. Increased Production of AGEs

**Figure 2:**
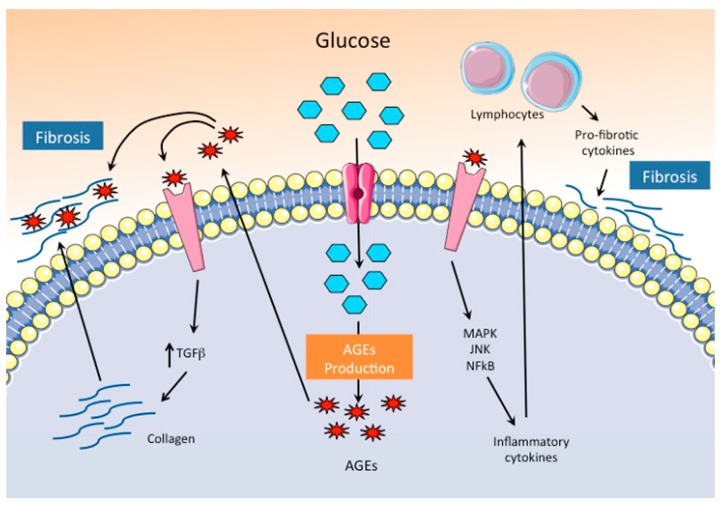
Pathogenic mechanisms of hyperglycemia through the production of AGEs. Hyperglycemia and inhibition of glycolysis favor the accumulation of glucose and the production of AGEs in cardiomyocytes. Activation of AGE–specific receptors (RAGE) signaling pathway increases collagen production and turns on intracellular inflammatory pathways, both of which enhance fibrosis.

AGEs mostly consist of proteins, but also lipids and nucleic acids that have been glycated by the non-enzymatic action of reducing sugars. Secondary to the hyperglycemia that characterizes uncontrolled diabetic patients, AGEs promote the crosslinking of collagen and other extracellular matrix proteins, impairing their normal degradation (Figure 2). The changes in the mechanical properties of the extracellular matrix proteins lead to increased fibrosis and myocardial stiffness [17]. Furthermore, the interaction of AGEs with specific receptors (RAGEs) leads to enhanced expression of collagen and other extracellular matrix proteins through the upregulation of TGFβ, contributing by this means to the exacerbation of the cardiac dysfunction induced by fibrosis. In addition, AGEs induce the expression of their specific RAGEs receptors, and generate a positive feedback loop that exacerbates the negative impact of AGEs on cardiac function. AGEs-RAGE signaling also activates a pro-inflammatory response through MAPK, JNK, and NF-kB that leads to the recruitment and activation of T helper lymphocytes and induces the production of pro-fibrotic cytokines, favoring interstitial fibrosis and impairing diastolic function [18]. The relevant contribution of AGEs to the etiopathogenesis of DCM has been demonstrated by the use of molecules with RAGE antagonistic activity, which were able to improve the systolic function and reduce the expression of pro-fibrotic proteins in a mouse of type 2 diabetes associated with obesity [19].

#### 3.1.2. Increased Substrate Flux through the Hexosamine Biosynthetic Pathway

Once into the cell, accumulated glucose can be diverted towards the hexosamine biosynthetic pathway. The hexosamine pathway provides the UDP-N-acetylglucosamine that serves as a donor for *O*-linked-β-*N*-acetylglucosamine (O-GlcNAc) protein modification. Post-translational O-GlcNAcylation of targeted proteins, including transcription factors, structural proteins or enzymes, leads to changes in their activity and function. Whereas the flux of glucose through the hexosamine pathway under non-pathological conditions must be considered as a regulatory mechanism of the cellular function in response to nutrients, the sustained activation of this pathway, as it occurs in diabetes, is known to have detrimental effects.

Increased accumulation of O-GlcNAcylated proteins has been observed in the heart of rodent models of type 1 and type 2 diabetes [20,21]. Several relevant cardiac proteins have been found to be O-GlcNAcylated, including phospholamban (a regulator of the calcium pump SERCA2a), calmodulin-dependent protein kinase II (CAMKII) or proteins of the contractile machinery (i.e., actin and troponin I) [22,23]. The O-GlcNAcylation of phospholamban and CAMKII is compatible with the contractile dysfunction observed in DCM that results from impaired calcium handling (see Section 3.1.6). On the other hand, O-GlcNAcylation of contractile proteins has been shown to alter their sensitivity to calcium, and by this means it contributes to the contractile dysfunction of the diabetic heart [24]. Although the negative effects on cardiomyocyte function of increased flux through the hexosamine biosynthetic pathways and the resulting protein O-GlcNAcylation have been demonstrated in vitro using cultured cardiac cells [25], compelling evidence that unequivocally demonstrates the contribution of the hexosamine pathway to DCM in vivo is still missing.

#### 3.1.3. Lipotoxicity

**Figure 3:**
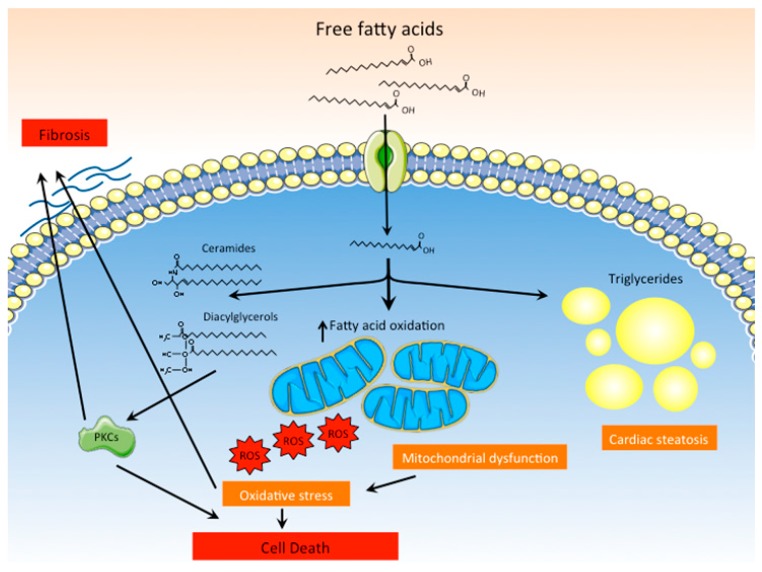
Lipotoxicity as a main pathogenic mechanism of DCM. Increased uptake of fatty acids by cardiomyocytes favors their accumulation as triglycerides within the cells. Moreover, inefficient oxidation of fatty acids by dysfunctional mitochondria leads to excessive production of ROS and cell death. Lipid intermediates and other lipid species, such as ceramides and diacylglycerols, also accumulate in cardiomyocytes and activate PKCs, promoting fibrosis and cell death.

As previously mentioned, DCM is often associated with the accumulation of lipids in the myocardium. The cardiac steatosis observed in diabetic patients and animal models of DCM is the result of increased fatty acid uptake and storage under the form of triglycerides (Figure 3). Intracellular lipid accumulation is unequivocally favored by the hyperlipidemic milieu that characterizes diabetes, which results from enhanced hepatic lipid synthesis and uncontrolled lipolysis in adipose tissues. Although triglyceride accumulation within cardiomyocytes is the most evident and detectable manifestation of intracellular lipid accumulation, lipotoxicity cannot be attributed to triglycerides themselves but to the accumulation of noxious lipid intermediates, such as ceramides, diacylglycerols or acylcarnitines [26,27]. The accumulation of these lipid species promotes mitochondrial dysfunction, endoplasmic reticulum stress or apoptosis by mechanisms that are not fully understood. For instance, ceramides and diacylglycerols have been shown to activate different protein kinase C isoforms, like PKCβ, PKCδ, or PKCθ. Activation of PKCs-mediated signaling pathways promotes inflammation, fibrosis or cell death, all of which have been shown to contribute to cardiac dysfunction [28]. Excessive lipid uptake by the myocardium also alters cardiomyocyte metabolism, favoring fatty acid oxidation in detriment of glucose as the main energetic substrate [29]. The increased flux of fatty acids into the mitochondria for their oxidation results in uncoupling of mitochondria and excessive production of ROS (see Section 3.1.4), which promote cell death and contribute to cardiac dysfunction [30].

#### 3.1.4. ROS Production and Mitochondrial Dysfunction 

Oxidative stress has been identified as a major contributing factor to the onset and development of DCM. Numerous studies in animal models of type 1 or type 2 diabetes and few others in humans have demonstrated the presence of oxidative stress in the diabetic heart. For instance, increased levels of hydrogen peroxide, which correlate with a high content of peroxidized lipids and nitrosylated proteins, as well as cell death by apoptosis, have been found in hearts of patients with type 2 diabetes [31,32]. Similarly, ROS-induced modification of lipids and proteins has been described in several rodent models of DCM, together with other markers of oxidative stress, such as altered expression levels of detoxifying enzymes (i.e., catalase, peroxidase) or gluthation [33,34,35,36,37]. In support of a relevant causative contribution of oxidative stress to the pathogenesis of DCM, reduction of ROS levels by the administration of antioxidants, such as dehydroepiandrosterone (DHEA) [33] to rodent models, as well as the ectopic overexpression of catalase [37] or superoxide dismutase [35], have been shown to reduce cardiac fibrosis and to improve cardiac contractile function.

It is necessary to remark that mitochondria are the major source of cellular ROS. It has been suggested that an impaired capacity of mitochondria to efficiently oxidize substrates, such as fatty acids, would favor the production of ROS. In this regard, numerous studies have comprehensively demonstrated that diabetic patients exhibit a reduction of mitochondrial mass and/or activity in multiple tissues, including skeletal muscle and adipose tissue (reviewed in [38]). The heart does not appear to be an exception and evidence of impaired mitochondrial function, such as a decrease in the PCr/ATP ratio, has been observed in diabetic patients, tightly correlating with diastolic dysfunction [39]. Impaired mitochondrial oxidative capacity has also been observed in several rodent models of type 1 or type 2 diabetes [15,30,35,40,41]. The primary cause of such mitochondrial dysfunction in diabetic individuals is unclear. Whereas some authors have reported a decrease in the expression of genes encoding for mitochondrial proteins of the OxPhos system [15,30], some others have reported the absence or even an increase in the expression of such genes [35,42]. Regardless of whether the mitochondrial oxidative machinery is reduced or not in diabetic individuals, numerous studies in rodent models have shown that the heart of diabetic individuals exhibits increased fatty acid uptake and oxidation, a metabolic signature of diabetic heart. The exacerbated fatty acid oxidation observed in diabetic hearts is, at least in part, the result of the increased levels of circulating fatty acids that characterizes the diabetic milieu. Still, the question is: How increased fatty acid oxidation results in mitochondrial dysfunction and increased ROS production? According to the data gathered from rodent models of diabetes, it is thought that mitochondria of diabetic hearts cannot handle the overwhelming amount of fatty acids that are delivered into the cardiomyocytes. This results in inefficient oxidation of lipids, the uncoupling of mitochondria and enhanced production of ROS [30,43]. In turn, the abnormally high levels of ROS produced within cardiac cells further favor mitochondrial dysfunction, aggravating the worsening of cardiac energetics and further reducing cardiac efficiency and function.

#### 3.1.5. Activation of RAS

Diabetes has been documented to suppress the circulating RAS, causing a decrease in the blood levels of renin and angiotensin II [44]. Paradoxically, local activation of the RAS pathway, independently of the global circulating RAS, has been described in several tissues of diabetic patients, including heart [45]. The cardiac activation of RAS has been suggested to contribute to cardiac dysfunction in individuals with DCM by promoting fibrosis, increased oxidative stress, and cardiomyocyte death.

**Figure 4:**
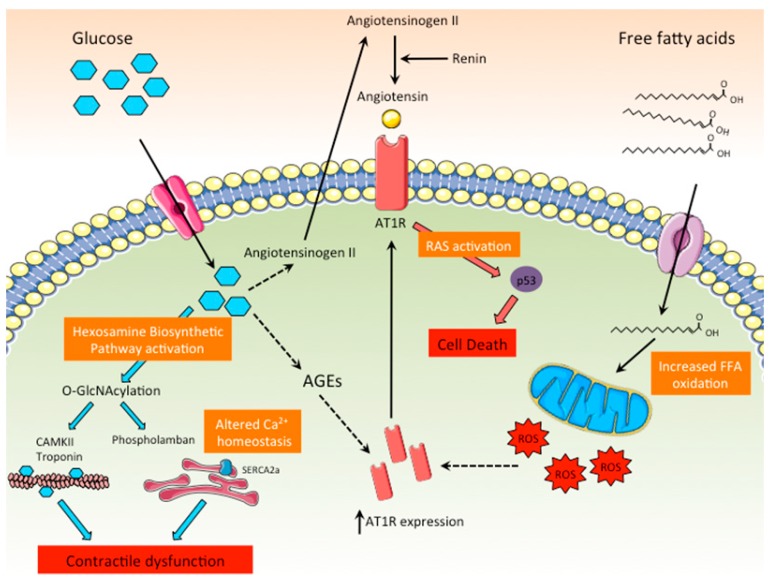
Role of the RAS and the hexosamine biosynthetic pathways in DCM. High intracellular glucose levels promote the activation of the hexosamine biosynthetic pathway, leading to the O-GlcNAcylation of key proteins related to the cardiac contractile function or involved in calcium homeostasis. In addition, high glucose levels directly stimulate the production of angiotensinogen II, leading to the activation of RAS in cardiomyocytes and cell death. AGEs and ROS promote the synthesis of the ATR1, further enhancing RAS activation.

Diabetes activates local RAS by different means (Figure 4). It has been demonstrated that hyperglycemia can directly stimulate the production of angiotensin II in cardiomyocytes, an effect that contributes to cardiomyocyte death by apoptosis through the activation of p53 [45,46]. In addition, diabetes exacerbates the expression of angiotensin receptor 1 (AT1R) in heart, amplifying the activation of RAS signaling in cardiomyocytes [47]. Other indirect mechanisms by which diabetes increases the activity of cardiac RAS are related to dyslipidemia and the exacerbated production of AGEs and ROS associated with diabetes, all of which stimulate the cardiac RAS pathway by up-regulating AT1R expression [48]. Although most of the experimental evidence linking abnormal RAS activation with DCM has been obtained in rodent models of diabetes, the significant improvement of left ventricle hypertrophy and systolic function in diabetic patients treated with the RAS antagonist aliskiren, a renin inhibitor, clearly supports the notion that RAS activation is a major contributor to the pathogenesis of DCM [49,50].

#### 3.1.6. Calcium Mishandling

Calcium is an essential regulator of muscle contraction. Upon cardiomyocyte depolarization, calcium enters the cell through the L-type calcium channels and promotes the release of more calcium from the sarcoplasmic reticulum. The large increase in intracellular calcium concentration triggers cellular contraction by binding to the contractile proteins. Subsequently, calcium returns to the sarcoplasmic reticulum through the action of several ion transporters, including the sarcoplasmic reticulum calcium pump (SERCA2a), the sodium–calcium exchanger and the sarcolemmal calcium ATPase.

Alterations in calcium homeostasis have been linked to the functional derangements found in heart of diabetic individuals. Studies in animal models of diabetes have reported that calcium release and reuptake by the sarcoplasmic reticulum is depressed in cardiomyocytes of diabetic individuals [51,52,53,54]. As a result, calcium transients are smaller and slower, altering the normal contractile activity of the heart. Several studies coincide in pointing at reduced activity of the SERCA2a pump, but also of others calcium transporters, such as the sodium–calcium exchanger or the sarcolemmal calcium ATPase, the expression of which is reduced in heart of diabetic individuals, as one of the major causes of the disturbances in calcium handling in DCM [55,56].

### 3.2. Effects of Insulin Resistance on Metabolic Flexibility and its Implications on Cardiac Dysfunction

Reduced or complete lack of insulin signaling is a hallmark of diabetes. Whereas in type 1 diabetic individuals insulin signaling is absent as a result of the lack of insulin synthesis and/or secretion, type 2 diabetes is characterized by impaired, but still active, insulin signaling. Regardless of the type of diabetes, altered insulin signaling in diabetic individuals leads to profound, but similar, alterations in metabolism, which is characterized by reduced insulin-dependent glucose uptake and utilization, and exacerbated usage of lipids as metabolic substrates. Altered metabolism directly impacts cardiomyocyte function, ultimately being accountable for most of the functional and structural alterations found in DCM.

The heart requires an enormous amount of energy to maintain its contractile function. Cardiac cells can use a wide variety of substrates as a source of energy, including amino acids and ketone bodies, although carbohydrates and lipids are their preferred substrates to obtain ATP via oxidative phosphorylation in mitochondria. Overall, a healthy heart predominantly obtains around 70% of the energy from fatty acids and 20–30% from glucose [57]. However, the use of carbohydrates or lipids varies along the day depending on substrate availability and is tightly controlled by hormones, like insulin and glucagon. During the feeding state, secretion of insulin inhibits adipose tissue lipolysis and promotes hepatic lipogenesis, decreasing the availability of fatty acids for the heart and other tissues. Simultaneously, insulin promotes glucose uptake and utilization by cardiomyocytes. Contrarily, in fasting conditions, the decrease in insulin levels and the rise in glucagon decrease glucose uptake and oxidation, while promoting fatty acid utilization. Therefore, metabolic flexibility, the capacity to switch from one substrate to another, is the main feature of a healthy heart.

The diabetic heart, however, is characterized by an unusual metabolic inflexibility. Indeed, the diabetic heart is unable to use glucose for the production of ATP and exclusively relies on fatty acids for such purpose. Such metabolic defect can be almost exclusively attributed to blunted insulin signaling. Thus, despite the hyperglycemia that characterizes the diabetic milieu, impaired insulin action results in reduced glucose uptake by cardiomyocytes through the insulin-sensitive glucose transporter GLUT4, which represents 70% of glucose transporters in the heart. Nevertheless, glucose gets into cardiomyocytes by other insulin-independent glucose transporters, such as the sodium glucose co-transporter SGLT1, whose expression is increased in diabetic individuals [58]. Intracellular glucose accumulation is also favored by a reduction in the glycolytic flux as a result of the inhibition of the pyruvate dehydrogenase complex (PDH) by pyruvate dehydrogenase kinase 4 (PDK4), whose expression and activity are dramatically increased in diabetic heart as a consequence of the negative action of insulin on *Pdk4* gene expression [59]. Increased intracellular accumulation of glucose, as mentioned in previous sections, is known to cause toxic effects by enhancing the formation of AGEs. Furthermore, high glucose intracellular concentration favors the rerouting of glucose into the hexosamine biosynthetic pathway, resulting in the O-GlcNAcylation of target proteins. As discussed earlier (see Section 3.1.1 and Section 3.1.2), the increased flux of glucose through the AGE and hexosamine pathways contributes to the functional derangements of the diabetic heart.

Insulin resistance or lack of insulin signaling in adipose tissue of diabetic individuals impairs the inhibition of triglyceride lipolysis by insulin, leading to high levels of circulating fatty acids. This, together with the incapacity of diabetic heart to use glucose as a substrate for the production of ATP, favors the uptake and oxidation of fatty acids. Still, as mentioned previously, the inability of cardiac mitochondria to deal with such an overwhelming amount of lipids results in lipotoxicity. The exacerbated lipid metabolism observed in DCM is not simply the result of increased lipid uptake and increased flux through oxidative pathways as a direct result of increased activity of catabolic enzymes, but it implies a complete reprogramming of the entire cellular metabolism through broad changes in the transcriptional programs that govern lipid metabolism. Indeed, gene expression profiling studies in hearts of *db/db* diabetic mice have revealed an overall increase in the expression of genes related to different aspects of lipid metabolism, including triglyceride hydrolysis, cellular transport and both peroxisomal and mitochondrial β-oxidation of fatty acids [42]. In addition, an increase in the expression of genes related to lipid synthesis has been also observed, which together with the increase in the expression of genes involved in fatty acids transport yields a plausible explanation for the cardiac steatosis that characterizes DCM. The metabolic reprogramming of diabetic hearts appears mediated at the transcriptional level by the activation of hormone nuclear receptors estrogen-related receptor γ (ERRγ) and Peroxisome Proliferator-Activated Receptor α (PPARα), the expression of which is increased in diabetic models of DCM [42,60]. Both ERRs and PPARα are well-known regulators of oxidative metabolism by regulating, among others, the expression of genes involved in fatty acid catabolism [61,62]. In support of PPARα playing a relevant role in the metabolic reprogramming of diabetic hearts, transgenic mice that overexpress PPARα specifically in heart show an increase in fatty acid oxidation rates that is accompanied by cardiomyocyte hypertrophy and contractile dysfunction [60,63]. On the other hand, a key role of ERRγ in DCM has been recently suggested by in vitro studies showing that adenoviral-mediated ectopic overexpression of ERRγ in mouse cardiomyocytes is sufficient to recapitulate most of the alterations found in diabetic hearts, including increased *Pdk4* expression, enhanced fatty acid oxidation, activation of the transcriptional program related to lipid metabolism, particularly lipid catabolism, and increased cardiomyocyte size [42]. Interestingly, in addition, ERRγ controls the expression of PPARα by directly binding to the *Ppara* promoter [42,64], highlighting the role of the ERRγ-PPARα axis in the metabolic reprogramming of the diabetic heart.

## 4. Rodent Models of Impaired Insulin Signaling for the Study of DCM

The studies aimed at unraveling the factors involved in the onset and progression of DCM have been based on the use of rodent models. Still, the use of cultured cells has been instrumental in precisely defining the mechanisms of action and pathogenic relevance of such mechanisms. The rat H9C2 and mouse HL-1 cell lines together with primary rat neonatal cardiomyocytes have been extensively used. However, these in vitro models are not exempt from severe limitations. Thus, whereas HL-1 cells have an atrial origin, H9C2 myoblasts are frequently used without any further differentiation into mature muscular cells, precluding in both cases any conclusion about the real impact of the results obtained on adult ventricular cardiomyocytes. More recently, the use of induced pluripotent stem cell (iPSC)-derived cardiomyocytes has proven to be very valuable for the study of DCM [65,66]. However, one of the major drawbacks of this model, but which can be extended to others cellular models, is the difficulty to accurately reproduce all the cellular and environmental features the define DCM, including glucotoxicity, lipotoxicity, and insulin resistance. Although this has been partially achieved by culturing iPSC-derived cardiomyocytes in media with high levels of fatty acids (i.e., palmitate) and glucose [67,68], the culture conditions are far from accurately reproduce the complex metabolic and hormonal features, as well as (dys)functional characteristics found in diabetic subjects. Therefore, the use of animal models still remains the best approach for the study of DCM. 

### 4.1. Genetic Models of Cardiac-Specific Disruption of Insulin Signaling for the Study of DCM

The use of genetically-engineered mouse models that lack specific components of the insulin signaling pathway has proven extremely useful to precisely define the contribution of impaired insulin signaling to DCM without the confounding effects derived from the systemic metabolic alterations found in genetic- or diet-induced models of insulin resistance/diabetes associated with chronic obesity. 

Mice lacking insulin receptor (IR) specifically in the heart show impaired cardiac function accompanied by decreased heart size, which results in part from a reduction in cardiomyocyte size [69,70]. The reduction in heart size found in heart-specific IR knockout mice highlights the relevance of proper insulin signaling in cardiac growth, beyond its effects on metabolism. At the metabolic level, however, loss of insulin signaling causes an unexpected increase in basal glycolysis rates, although both glucose and fatty acid oxidation are reduced. The loss of IR results in a reduction in GLUT1 but, surprisingly, such loss is accompanied by an increase in GLUT4, which is apparently sufficient to compensate for impaired glucose transport in the absence of proper insulin signaling. Contrary to what it has been observed in rodent models of obesity-associated type 2 diabetes or streptozotocin-induced type 1 diabetes (see Section 4.2), mice lacking IR exhibit a reduction in the expression of fatty acid oxidation genes (*Mcad*, *Lcad*, *Vlcad*, *Ppara*), as well as a reduction in the expression of *Pdk4*, what would explain the reduced fatty acid oxidation rates found in these mice [69].

Knockout mice with simultaneous deletion of genes encoding for insulin receptor substrate-1 and -2 (IRS-1 and IRS-2) specifically in heart show a phenotype similar to that of heart-specific IR knockout mice. Far from showing cardiac hypertrophy, cardiac-specific IRS1/2 knockout mice develop dilated cardiopathy, characterized by increased diameter of cardiac chambers and thinned cardiac walls [71]. Loss of IRS1/2 leads to increased cardiomyocyte death and interstitial fibrosis that contribute to depressed cardiac function and early death by two months of age. Consistent with lack of proper insulin signaling and contrary to what was described for heart-specific IR knockout mice, cardiac-specific IRS1/2 knockout mice exhibit a reduction in the expression of genes related to glucose metabolism (i.e., *Glut1*, *Glut4*, *Gck*) and an increase in the expression of *Pdk4*. However, in contrast with the characteristic gene expression pattern of DCM, the expression of genes related to lipid catabolism (i.e., *Vlcad*) was reduced [71], what is suggestive of a decreased fatty acid oxidation in the absence of IRS1/2.

3-phosphoinositide-dependent kinase-1 (PDK1) is another key component of the insulin signaling pathway that is required for the activation of AKT/PKB. Mice lacking PDK1 specifically in the heart show a phenotype very similar to the one shown by the models described earlier. Cardiac-specific PDK1 knockout mice die around 5–11 weeks of age from severe heart failure [72]. Hearts from mice lacking PDK1 exhibit clear signs of wall thinning accompanied by interstitial fibrosis and reduced cardiomyocyte size. To overcome the early lethality of the cardiac-specific PDK1 knockout mice, Ito and collaborators generated a tamoxifen-inducible cardiac-specific PDK1 knockout mouse model [73]. Lack of PDK1 during adulthood leads to death from heart failure 5–15 weeks after *Pdk1* deletion. Wall thinning and chamber dilation, together with increased fibrosis and cardiomyocyte apoptosis were developed soon after *Pdk1* was deleted [73]. Unfortunately, how metabolism is affected by the lack of PDK1 in those mice has not been addressed.

### 4.2. Rodent Models of Diabetes or Systemic Insulin Resistance for the Study of DCM

The mouse models defective in specific components of the insulin signaling pathway in cardiac tissue highlight the relevance of insulin for proper cardiac function and development, as well as in cardiomyocyte growth and survival. However, complete lack of insulin signaling specifically in the heart does not always phenocopy all the cellular and molecular derangements found in DCM. This suggests that DCM is the result of complex interactions between cardiac cells in which insulin signaling is impaired and the diabetic milieu that results from insulin resistance or absence of insulin signaling in other tissues. In this regard, the use of rodent models of diabetes or systemic insulin resistance has proven very valuable for the study of DCM. The use of such rodent models has been extensively reviewed elsewhere [15] and, therefore, here, we will only highlight some of the main features of the most commonly used models.

Streptozotocin (STZ)-induced type 1 diabetes rat or mouse models have been frequently used for the study of DCM. The toxic effects of STZ on pancreatic β-cells result in loss of β-cells, with the consequent deficiency in insulin secretion, despite insulin sensitivity being maintained in peripheral tissues. STZ-induced diabetes rodent models exhibit cardiac dysfunction, the severity of which is proportional to the time of diabetes evolution. Both systolic and diastolic left ventricular dysfunction has been reported in some studies, associated with reduced ejection fraction and/or reduced rate of relaxation [74,75,76]. However, other studies have reported no major alterations in heart function [77]. Nevertheless, STZ-induced type 1 diabetic mice and rats show most of the cellular and metabolic features described in DCM, including cardiomyocyte hypertrophy and death, oxidative stress, fibrosis, decreased cardiac efficiency, altered calcium homeostasis and increased expression of genes related to fatty acid uptake and oxidation [33,45,60,75,76,78].

Rodent models of severe insulin resistance and type 2 diabetes associated with obesity as a result of a defect in the leptin signaling pathway have been also extensively used for the study of DCM. The *ob/ob* mice show a recessive mutation in the gene encoding for leptin, an adipose-specific anorexigenic hormone, whereas the *db/db* mice -and their equivalent Zucker diabetic fatty (ZDF) rats- are characterized by a point mutation in the leptin receptor gene. As a result of the disruption in leptin signaling, *ob/ob* and *db/db* mice and ZDF rats are hyperphagic and develop severe obesity, insulin resistance and type 2 diabetes. Although both models show impaired leptin signaling, a major difference between *ob/ob* and the *db/db* mice is the temporal course of diabetes, which develops earlier in *db/db* mice. This might be responsible for the subtle differences found between *ob/ob* and *db/db* mice with regard to the cardiac outcome. In addition, the technical approaches used in the different studies to evaluate cardiac structure and function could be blamed for the variable, sometimes even contradictory, results obtained in different studies using the same mouse model. For instance, echocardiographic studies have yielded very variable results with regard to cardiac structure and function. Thus, whereas a reduction in left ventricular mass and decreased diameter of the ventricular cavities, both at systole or diastole, have been reported by some studies in *db/db* mice [42], others researchers have reported an increased in the left ventricle internal cavity diameter without major changes in ventricular mass [41,79] or the lack of noticeable and consistent structural alterations [80,81,82,83]. Contrarily, the assessment of cardiac function in isolated working hearts unequivocally revealed altered cardiac function in hearts of *ob/ob* and *db/db* mice, both of which exhibit a decrease in left ventricle systolic pressure and cardiac output (measured as aortic flow) and an increase in left ventricle developed pressure [29,30,43,55,84,85,86]. Moreover, *ob/ob* and *db/db* mice and ZDF rats accurately reproduce all the cellular and metabolic traits that define DCM, including cardiomyocyte hypertrophy, cardiac steatosis and cell death [27,42,80,81]. At the metabolic level, type 2 diabetic rodent models show metabolic inflexibility, characterized by reduced glucose oxidation and increased fatty oxidation and oxidative stress [29,43,84,85,86]. Consistent with the increase in fatty acid oxidation, these rodent models exhibit increased expression of genes involved in lipid uptake, mobilization, and oxidation [42,43,60,85].

## 5. Conclusions

From the numerous epidemiologic studies that show a steady rise in the prevalence of diabetes, it would not be too daring to predict that the prevalence of DCM will also escalate proportionally in the upcoming years. Our comprehension of the pathogenic mechanisms of DCM remains limited, in part due to the difficulty to diagnose the disease at the early stages of its development and also as a consequence of the scarcity of studies carried out in diabetic patients. Most of our knowledge about the pathogenic factors involved in DCM has been provided by studies performed in a variety of rodent models of type 1 and type 2 diabetes or genetically engineered mouse models that lack important molecular components of the insulin signaling pathway. These studies have identified mitochondrial dysfunction, increased AGEs production, oxidative stress, calcium mishandling, lipotoxicity, enhanced flux through the hexosamine biosynthetic pathway and activation of the RAS pathway as factors that trigger or contribute to the structural and functional alterations of cardiac cells. Because these pathogenic factors may concur in the same patient, the use of specific drugs to target one of these components of the disease might not be sufficient to revert or prevent heart failure. However, compelling evidence obtained from the studies in rodent models supports the notion that the factors that mediate the cellular derangements in diabetic hearts are the direct result of impaired insulin signaling in cardiomyocytes. Therefore, improving insulin signaling in the heart appears as a more effective strategy for the integral treatment of DCM.
